# Data preprocessing workflow for exhaled breath analysis by GC/MS using open sources

**DOI:** 10.1038/s41598-020-79014-6

**Published:** 2020-12-15

**Authors:** Rosa Alba Sola Martínez, José María Pastor Hernández, Gema Lozano Terol, Julia Gallego-Jara, Luis García-Marcos, Manuel Cánovas Díaz, Teresa de Diego Puente

**Affiliations:** 1 Biotechnology Group, Department of Biochemistry and Molecular Biology and Immunology (B), Faculty of Chemistry, University of Murcia, Campus of Espinardo, Regional Campus of International Excellence ‘‘Campus Mare Nostrum’’, P.O. Box 4021, 30100 Murcia, Spain; 2 Biomedical Research Institute of Murcia (IMIB-Arrixaca), Murcia, Spain; 3 Respiratory and Allergy Units, Arrixaca Children’s University Hospital, University of Murcia, Murcia, Spain; 4 Network of Asthma and Adverse and Allergy Reactions (ARADyAL), Health Institute Carlos III, Madrid, Spain

**Keywords:** Biochemistry, Computational biology and bioinformatics, Systems biology, Biomarkers, Diseases, Medical research

## Abstract

The noninvasive diagnosis and monitoring of high prevalence diseases such as cardiovascular diseases, cancers and chronic respiratory diseases are currently priority objectives in the area of health. In this regard, the analysis of volatile organic compounds (VOCs) has been identified as a potential noninvasive tool for the diagnosis and surveillance of several diseases. Despite the advantages of this strategy, it is not yet a routine clinical tool. The lack of reproducible protocols for each step of the biomarker discovery phase is an obstacle of the current state. Specifically, this issue is present at the data preprocessing step. Thus, an open source workflow for preprocessing the data obtained by the analysis of exhaled breath samples using gas chromatography coupled with single quadrupole mass spectrometry (GC/MS) is presented in this paper. This workflow is based on the connection of two approaches to transform raw data into a useful matrix for statistical analysis. Moreover, this workflow includes matching compounds from breath samples with a spectral library. Three free packages (*xcms*, *cliqueMS* and *eRah*) written in the language R are used for this purpose. Furthermore, this paper presents a suitable protocol for exhaled breath sample collection from infants under 2 years of age for GC/MS.

## Introduction

Over the last decades, the analysis of volatile organic compounds (VOCs) in exhaled breath has been proposed as a promising approach to searching for biomarkers for the diagnosis and monitoring of different diseases, and has been used to check for pollution exposure or smoking activities^1–7^. One of the main advantages of this strategy is that it is based on a noninvasive procedure^1^. This is especially important for populations such as children and elderly people and for diseases whose current standard diagnoses use invasive techniques such as biopsies and bronchoscopies^8–10^. However, this approach is still in the biomarker discovery phase and has not yet been implemented in clinics^11,12^. Several steps need to be overcome to achieve clinical utility as a biomarker. In general, this process involves biomarker discovery, analytical validation, and clinical validation^13^. The biomarker discovery phase workflow includes study design, breath sampling, analysis of exhaled breath, data preprocessing, identification of VOCs, data analysis, interpretation of results and putative biomarker validation in an independent cohort study^14^.

Currently, online real-time breath analysis is emerging through technologies based on mass spectrometry (MS), such as SESI-HRMS (secondary electrospray ionization – high-resolution MS), SIFT-MS (selective ion flow tube mass spectrometry), or PTR-MS (proton transfer reaction mass spectrometry)^15^. Nevertheless, the most widely used technology for VOC analysis in exhaled breath is currently MS coupled with gas chromatography (GC/MS), which is a highly sensitive and reliable technique. This technique is used mainly in offline breath analysis^11,16,17^. Preconcentration methods such as sorbent-containing thermal desorption (TD) tubes and solid phase microextraction (SPME) are required in offline breathing analysis^18^. In GC/MS analysis, the compounds present in exhaled air are fragmented into ions with different mass to charge ratios (*m/z*)^19^. Although both electron ionization (EI) and chemical ionization (CI) can be used in GC/MS, EI at 70 eV is clearly the most popular. EI is a hard ionization methodology that has high reproducibility. Therefore, comparisons with mass spectral libraries and interlaboratories are allowed^20^. On the other hand, there are two approaches of data acquisition in GC/MS: acquisition of all ions within a certain range of *m/z* (full scan mode) and acquisition of a unique ion (selected ion monitoring (SIM) mode). The SIM mode is frequently used in targeted analysis, which consists of the determination of a few known compounds. In contrast, the full scan mode is generally used for untargeted analysis, concerning any compound present in a sample^21,22^. Hence, untargeted analysis is widely employed in the biomarker discovery phase^23^.

Preprocessing the vast amount of raw data after the analysis of samples by TD-GC/MS for the correct quantification and identification of VOCs present in samples is essential before performing statistical analyses^24,25^. However, this step is often poorly reported in studies about VOC analysis in exhaled breath^26^. Therefore, the development of reproducible and comparable protocols for data preprocessing plays a crucial role in overcoming the discovery phase^12,26^. The scientific community remains stalled regarding the use of proprietary software, despite the great advances in recent years in the field of bioinformatics, especially in open source solutions. Indeed, currently, some packages based on the R programming language and computational tools are freely available for this task^23,27–30^.

The typical challenges in data preprocessing are: large volumes of raw data (especially in untargeted analysis), background noise, variations in retention times of VOCs between samples, variations in mass spectrum profile of VOCs (variations in relative intensities values of features within a compound) between samples, or overlapping of VOCs with very similar retention times, among others^24,27,31,32^. At present, the strategies to carry out the preprocessing of volatilome data are classified into two main groups. Basically, the first approach focuses mainly on finding the ion peaks detected in an EI-MS spectrum (features) or so-called peak-picking, and the second approach aims to determine the compounds of breath samples by a spectral deconvolution process. On the one hand, the difficulty in elucidating the identity of compounds is a weakness of the first approach^27^, and on the other hand, the use of deconvolution is questionable because it may add errors and increase variation^25^. Therefore, this paper describes a new and simple procedure for preprocessing data after exhaled breath analysis by a couple system of thermal desorption and gas chromatography—single quadrupole mass spectrometry (TD-GC/q-MS) that integrates the two approaches. Thus, as a result, it is possible to benefit from the advantages of both strategies, including verification to reduce possible errors and avoid compounds duplications (detection of a single compound as two compounds). For this purpose, the functions of three packages based on the R programming language are implemented: *xcms*^23^, *cliqueMS*^28^ and *eRah*^27^.

## Results

### Workflow of data preprocessing

**Figure 1:**
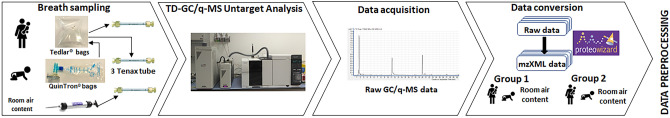
Steps of the biomarker discovery phase conducted before data preprocessing.

**Figure 2:**
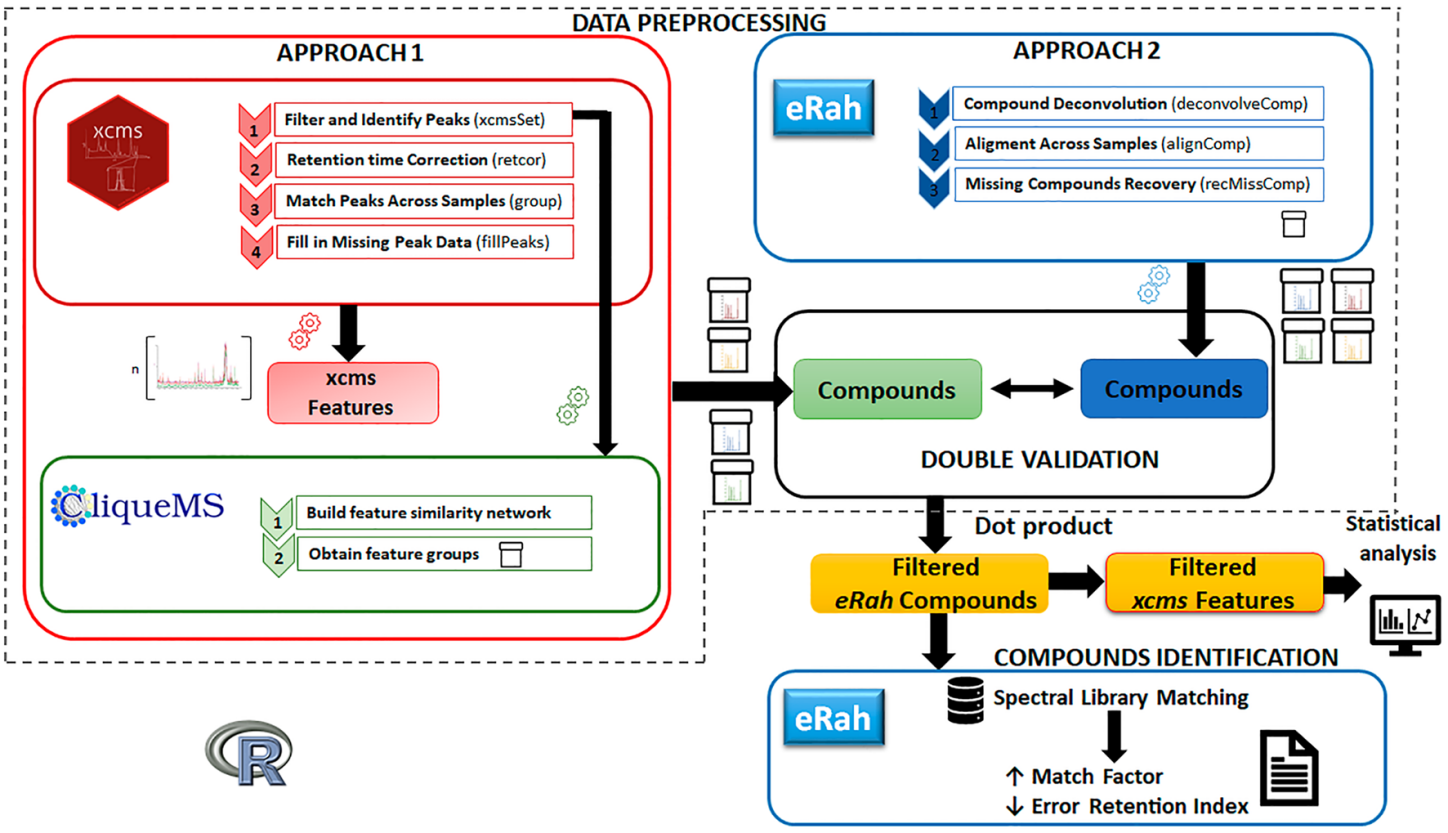
Workflow for data preprocessing after exhaled breath analysis using TD-GC/q-MS.

The guidelines for the raw data acquisition process performed before data preprocessing are shown in Fig. 1. The raw data acquired after analysis of exhaled air samples from mother–child pairs of the NELA (nutrition in early life and asthma) birth cohort (see the Methods section for details) were used for developing the data preprocessing workflow. As can be observed in Fig. 1, the step prior to data preprocessing was the conversion of raw data to an open standard format such as mzXML. For this purpose, the open source program *MSConvert* from *Proteowizard* was used^33,34^. Then, the samples were randomly divided into two groups (Group 1 and Group 2). In each group, samples from mothers, babies and the air content in the room were preprocessed separately. The workflow conducted for the preprocessing of data obtained by TD-GC/q-MS is shown in Fig. 2. Herein, searching of the compounds in the breath samples was carried out by the current two main approaches mentioned in the introduction: ion peaks detection and compounds detection. For the first approach, several packages of the free statistical software R (*xcms*^23^ and *cliqueMS*^28^) were used, whereas for the second approach, another package of R, *eRah*^27,35^, was applied. Finally, the two approaches were merged in the last step. A schematic overview of workflow and R-code of the most important functions are shown in Supplementary Tutorial.

**Figure 3:**
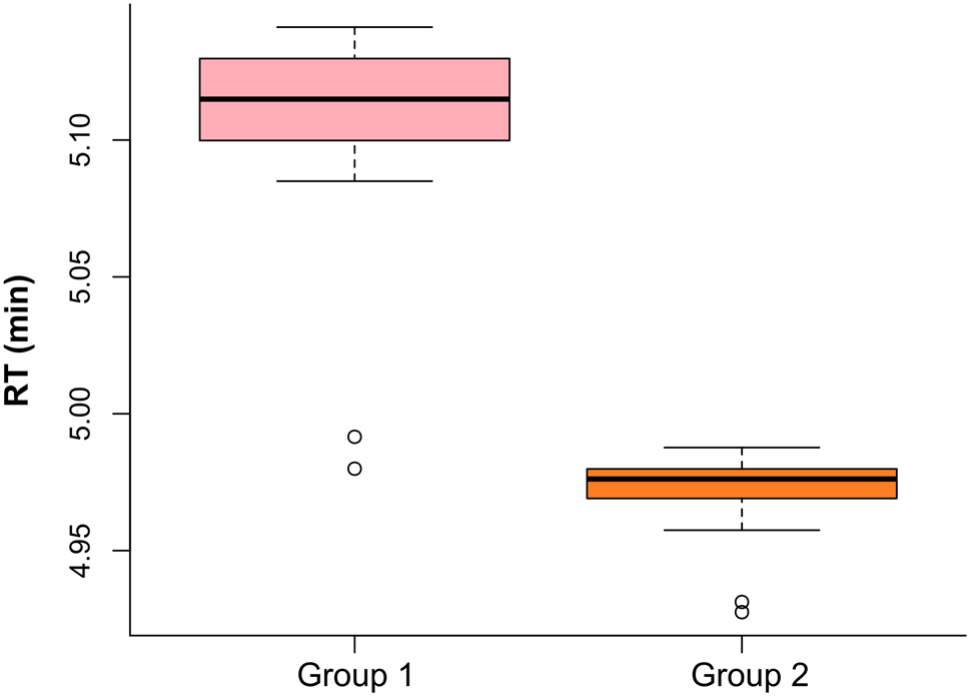
Differences in retention time between both sample groups. The graph shows the retention times of the 337 peaks detected in the room air content samples (210 in Group 1 samples) and (127 in Group 2 samples) at 91 m*/z* relative to toluene. Statistically significant differences between the retention times of both groups were observed using the two tailed Mann–Whitney U test (*p* value = 2.2e−16).

The procedure was as follows: first, breath samples of Group 1 were used to select the parameters included in each package function and for the design of the workflow; then, this workflow was performed on the breath samples of Group 2. The only difference between the groups was that the breath samples of the second group were analyzed with a different gas chromatography column than that used for Group 1. Although both columns were of the same brand and model, slight differences were observed between retention times of both the features and compounds of the samples of both groups. Figure 3 shows the differences between the retention times of a typical toluene peak for both groups, which was a ubiquitous compound in room air content samples. Since the objective was to verify the reproducibility of this protocol on another set of samples, no analysis parameters were altered to decrease the differences in the retention times. Several artifacts were identified in the samples, such as N,N-dimethylacetamide and phenol, which are commonly known and well-documented contaminants from Tedlar bags^36^ (as an example, Supplementary Fig. S1). Thus, both compounds were ubiquitous in all breath samples. Other known analytical artifacts, including polydimethylsiloxanes, were also identified.

Time-consuming for data preprocessing and compounds identification using this workflow was approximately 2 min per sample. Supplementary Table S1 details time spent in each phase of the workflow on each subset of samples from both groups.

#### Data preprocessing approach 1

The four main steps of *xcms* were conducted on our data with the following functions: *xcmsSet* (peak detection), *retcor* (retention time alignment), *group* (peak matching) and *fillPeaks* (missing peak filling) (Fig. 2). Before peak detection, denoising (smoothing) and baseline correction were performed to optimize the fwhm parameter of the *xcmsSet* function^23^. In peak detection (step 1), the goal was to determine the largest number of peaks in each sample without incorrectly duplicating peaks and with the separation of overlapping peaks. A couple of algorithms (MatchedFilter and Centwave) can be used for peak detection by *xcms*. Centwave decreases the computational cost of preprocessing and the data size and therefore is ideal for centroid high-resolution MS data. Profile MS data can also be transformed into centroid MS data. However, since our raw data were profile MS data acquired by a low-resolution mass spectrometer (single quadrupole), matchedFilter was chosen as the peak-picking algorithm for the extraction of the ion signals. In step 2, the *retcor* method was used to correct the differences between the retention times of different samples. This second step was required to ensure that the retention times were aligned across all samples in each subset because an intragroup variation in peak retention time was observed (Fig. 3). Then, peaks previously detected in each sample were grouped across all the samples in peak matching (step 3). Thus, peaks with similar characteristics (same *m/z* and similar retention time) were considered to be the same feature. A data matrix with the intensities of the features in each sample was obtained after performing steps 2 and 3.

Finally, the purpose of the last step was to fill in the gaps in this data matrix (step 4). The raw data were searched to determine the missing values of the intensities of the most frequent features in the samples between the start and end times of each retention time alignment feature (step 2)^23,37^.

The parameters selected for data preprocessing using *xcms* are described in Supplementary Table S2. The maximum intensity or "maxo" was used as the intensity value for each feature. Table 1 shows the total number of features detected in the samples of both groups by *xcms* preprocessing.

**Table 1:** Results obtained by both approaches.

Samples	Approach 1				Approach 2	
	*xcms*		*cliqueMS*		*eRah*	
	No of features		No of compounds		No of compounds	
	Group 1	Group 2	Group 1	Group 2	Group 1	Group 2
Exhaled breath of mothers	542	524	1613	1241	835	554
Exhaled breath of children	467	543	1242	1105	802	569
Room air content	867	937	2927	2223	913	610

Subsequently, *CliqueMS* was used to identify the features belonging to the same compound. The results obtained after performing *xcmsSet* (function of *xcms* for peak detection) were used as input data. The *CliqueMS* program workflow is described well in Senan et al*.*^28^. The grouping of features by *CliqueMS* is based on the construction of similarity networks and involved two steps: 1) build feature similarity network and 2) obtain feature groups. A disadvantage of this package is that it annotates samples one by one. To apply it to all samples, a loop from the features of the first step of *xcms* was programmed. The total number of different compounds identified in all samples from both groups is detailed in Table 1, and the parameters of *cliqueMS* used are compiled in Supplementary Table S2.

#### Data preprocessing approach 2

**Figure 4:**
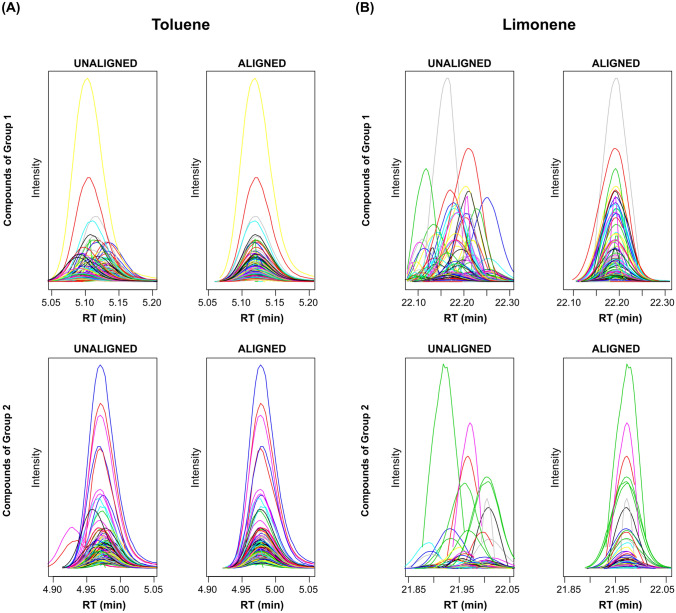
Spectral alignment using *eRah*. (**A**) Elution profile of toluene in room air content samples from both groups of samples (Group 1 and Group 2) before and after alignment. (**B**) Elution profile of limonene in room air content samples from both groups of samples (Group 1 and Group 2) before and after alignment. Plots of this figure were generated by the *plotAlign* function of package *eRah* in R.

In the second approach, three functions of *eRah*, *deconvolveComp*, *alignComp* and *recMissComp*, were used to perform three steps: 1) spectral deconvolution, 2) spectral alignment and 3) missing compound recovery (Fig. 2). Denoising and baseline correction were carried out automatically using the noise threshold parameter of the *deconvolveComp *function. The spectral deconvolution (step 1) of *eRah* includes two stages: i) compound match by local covariance (CMLC) and ii) orthogonal signal deconvolution (OSD). CMLC facilitates the identification of compounds in the chromatograms of samples, and OSD facilitates the retrieval of a compound spectrum given a compound elution profile^27^. A list with the *m/z* and relative intensities of ions that were part of the compounds was provided with the spectral deconvolution (step 1). Then, the most intense ion was assigned an abundance of 1000, and it was referred to as the base peak. Subsequently, alignment of the retention times of compounds across all samples (step 2) and the determination of missing compounds (step 3) were carried out. Although these two steps (2 and 3) had the same goal as the first approach, *eRah* uses a methodology based on other mathematical principles and different input data. As a result of step 2, the elution profiles of two ubiquitous compounds in the room air content samples (toluene and limonene) before and after alignment are depicted in Fig. 4. The function parameters selected for the second approach are shown in Supplementary Table S2. Since analysis by TD-GC/q-MS was performed using three different temperature ramps, the data preprocessing of samples by *eRah* was independently conducted in three parts (Supplementary Fig. S2).

Finally, the identification of compounds by spectral library matching was carried out using another *eRah* function. Only the compounds with a match factor (spectral similarity score) higher than 80% met the match criteria of compounds from the NIST (National Institute of Standards and Technology) library. Table 1 shows the total number of selected compounds using the second approach applying this filter.

#### Connecting both strategies

Both approaches were connected to perform a verification, avoiding duplicates and obtaining a unique list of filtered features and filtered compounds. Once the compounds of the samples were detected by the two approaches, a couple of steps were carried out to allow comparison between the compounds. First, since the second approach can determine the profile of each compound, the features detected with *xcms* that matched the ions of the compounds found with *eRah* were determined. For this purpose, a feature obtained by *xcms* and an ion of a compound obtained by *eRah* should have the same *m/z* signal and the same retention time ± 0.05 min to be considered the same. Second, the relative intensity of each feature within a compound obtained using *cliqueMS* was determined following a three-step protocol: 1) The average intensity of each feature was calculated across the whole set of samples. 2) A value of 1000 was assigned as the relative intensity value of the feature with the highest intensity in the compound (base peak). 3) The relative intensity value of the remaining features was calculated as follows: (average intensity of the feature/average intensity of the feature with the highest intensity) * 1000.

Afterwards, each compound obtained using *xcms-cliqueMS* was compared with all the compounds obtained by *eRah*. For this purpose, two vectors (one vector for a compound obtained by the first approach and another for a compound of the second approach) were constructed with 412 positions, where each position corresponded to an *m/z* signal within the range of *m/z* 38–450. These vectors were filled with the relative intensities (ranging from 0–1000) of ion fragments of each compound. A value of 0 indicated that the *m/z* signal was not detected in the compound. The dot product between both vectors was calculated, and a single value between 0–1 was obtained. A value close to 1 indicated a high level of similarity between the compounds. Therefore, it was possible to determine which *eRah* compound was the most similar to the *xcms-cliqueMS* compound (compound with the highest dot product value). This process is similar to comparing the spectrum of a compound with those of a library^38^. As a result, the compounds determined by both approaches were matched by a dot product. Then, only the *eRah* compounds detected whose dot product result was the highest for some *cliqueMS* compounds with a value higher than 0.7 were selected (filtered compounds). Moreover, a second filter was applied. Only the features obtained by *xcms* that had been previously matched with the filtered compounds of the second approach were selected (filtered features). Table 2 shows the total number of filtered features and filtered compounds of both groups. Furthermore, the feature intensity values were normalized across all the samples from each subset. The normalized intensity value from a feature in a sample = (log_10_ (intensity value from a feature in a sample)/( log_10_ (intensity values of a feature across all samples of each subset)) *1000.

**Table 2:** Filtered features and filtered compounds.

Samples	No of filtered features		No of filtered compounds	
	Group 1	Group 2	Group 1	Group 2
Exhaled breath of mothers	494	452	194	150
Exhaled breath of Children	377	483	155	158
Room air content	824	877	231	193

**Figure 5:**
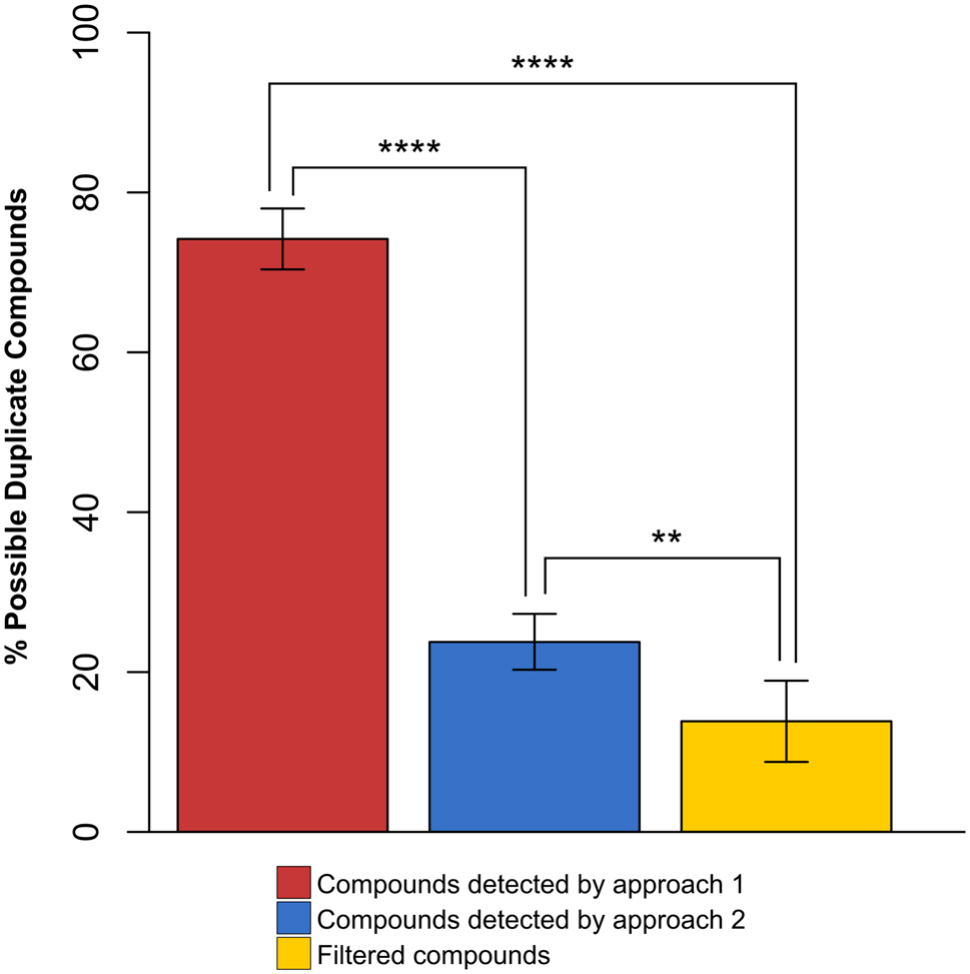
Possible Duplicate Compounds. Statistical comparisons between percentages each subset of possible duplicate compounds in compounds detected by first approach, compounds detected by second approach and filtered compounds were performed with two tailed ANOVA test followed by Bonferroni post-hoc tests (*p* value < 0.01(**), *p* value < 0.0001 (****)).

Among the compounds determined by *eRah* and *xcms-cliqueMS*, many possible duplicate compounds were found. However, after integration of both strategies, percentage of possible duplicate compounds was significantly reduced (Fig. 5). All the compounds detected in each subset were compared with each other by means of a dot product of the vectors of relative intensities of ion fragments of each compound. A possible duplication in *eRah* compounds was considered if two compounds had a dot product value higher than 0.8, the same retention time ± 0.05 min and its areas obtained by spectral deconvolution were correlated (Pearson correlation coefficient > 0.75). Duplications in *xcms-cliqueMS* compounds were determined based on retention time and dot product value.

### Identification of compounds

The *eRah* package can match the filtered compounds with the compounds registered in a spectrum library. *eRah* computes two factors to indicate the similarity between two compounds: the match factor and retention index error (RI error). While the first is related to the similarity between spectra, the other is related to the retention times of the compounds. Both values are expressed in percentages. Thus, if two compounds are identical, they have a 100% match factor (high similarity between spectra) and a 0% retention index error (minimum difference between retention times). In this data preprocessing workflow, the NIST library was selected as the spectrum library for matching. The RI error of each compound was calculated using reference compounds (with a retention time and RI value) and compared with the RI of the library compounds. The RI error of the filtered compounds was also determined in three independent parts since the analysis by TD-GC/q-MS was conducted using three temperature ramps (Supplementary Fig. S2). A VOC standard containing 54 individual compounds was used as the retention time reference for the calculated RI error. Only 10 compounds included in the mixture standard had RI available in the NIST library (Supplementary Table S3). In addition, none of these 10 compounds had a retention time within a range of 38.67–46.12 min (third temperature ramp). Although the NIST library is the largest spectrum library to our knowledge, its retention index database is very limited.

To increase the population of the retention index values for reference compounds, a commercial n-alkane standard mixture of C7-C30 saturated alkanes was used. The linear alkane retention times observed from each column are shown in Supplementary Table S4. Furthermore, the retention times of the linear alkanes were determined in the exhaled breath samples of both groups by comparing the retention times with those of known standards. For this purpose, ten samples of exhaled air from mothers were selected from Group 1 and were analyzed by the second approach. The matching of the *eRah* compounds was performed with the NIST library entries for searching exclusively for linear alkanes with 7–24 carbons. First, only the detected compounds with a match factor greater than 95% (high spectral similarity) were selected. Thus, among all the compounds detected by *eRah* in the samples, the 38 compounds showed this degree of spectrum similarity with any linear alkane. Then, these 38 compounds were independently matched to each of the linear alkanes. Thus, the compounds with a large match factor for a particular linear alkane or those with up to 5 points less than the largest match factor were selected. In addition, the compounds without the *m/z* equivalent to the molecular weight of the linear alkane were not included in the profile determined by *eRah*. Hence, 82 combinations of possible linear alkanes were obtained since each of the 38 compounds previously selected could match several linear alkanes. A search for linear alkanes was also carried out using *xcms* to reduce the number of combinations of possible linear alkanes. Thus, 76 features with *m/z* equal to the molecular weight of linear alkanes were selected. Afterwards, the two approaches were connected again. Then, only combinations of possible linear alkanes that had a RT ± 0.05 min compared with the RT of the feature with *m/z* equivalent to the molecular weight of the linear alkane were chosen. In this way, the possible combinations of alkanes were substantially reduced from 82 to 14. The compound with the lowest RT was also selected for each linear alkane. Three outliers were removed after they were observed when the RTs of the selected compounds and the number of carbons of the linear alkanes were plotted. Therefore, the retention times of 8 linear alkanes were ultimately determined (Supplementary Fig. S3). In addition, this process was replicated for 10 mother samples from Group 2. As seen in Supplementary Fig. S4, the linear alkane retention times determined for the exhaled air samples were very close to the retention times obtained from the commercial standard analysis.

#### Matching with NIST library

Finally, the following protocol was carried out to match every filtered compound with the NIST library. First, the threshold for the NIST match factor was set above 80%. Then, only those up to 5% below the largest match factor (always above 80%) were selected. A maximum of 450 entries were extracted for each filtered compound. Then, the RI of each filtered compound was calculated by *eRah* using the RT of reference standards (10 compounds from the VOC standard and linear alkane mixture). In addition, the RI error was calculated for all the extracted entries. Then, entries with an RI error greater than 20% were discarded. Finally, the entry with the lowest RI error and highest match factor was considered the best match for that filtered compound. However, if there was no RI error value below 20%, the entry with the highest match factor was considered an optimal match. As mentioned above, RI is not included in all NIST library entries. Supplementary Fig. S5 shows the percentage of matching of the filtered compounds from the NIST library, including the RI error and match factor values.

### Explorative analysis of filtered features

**Figure 6:**
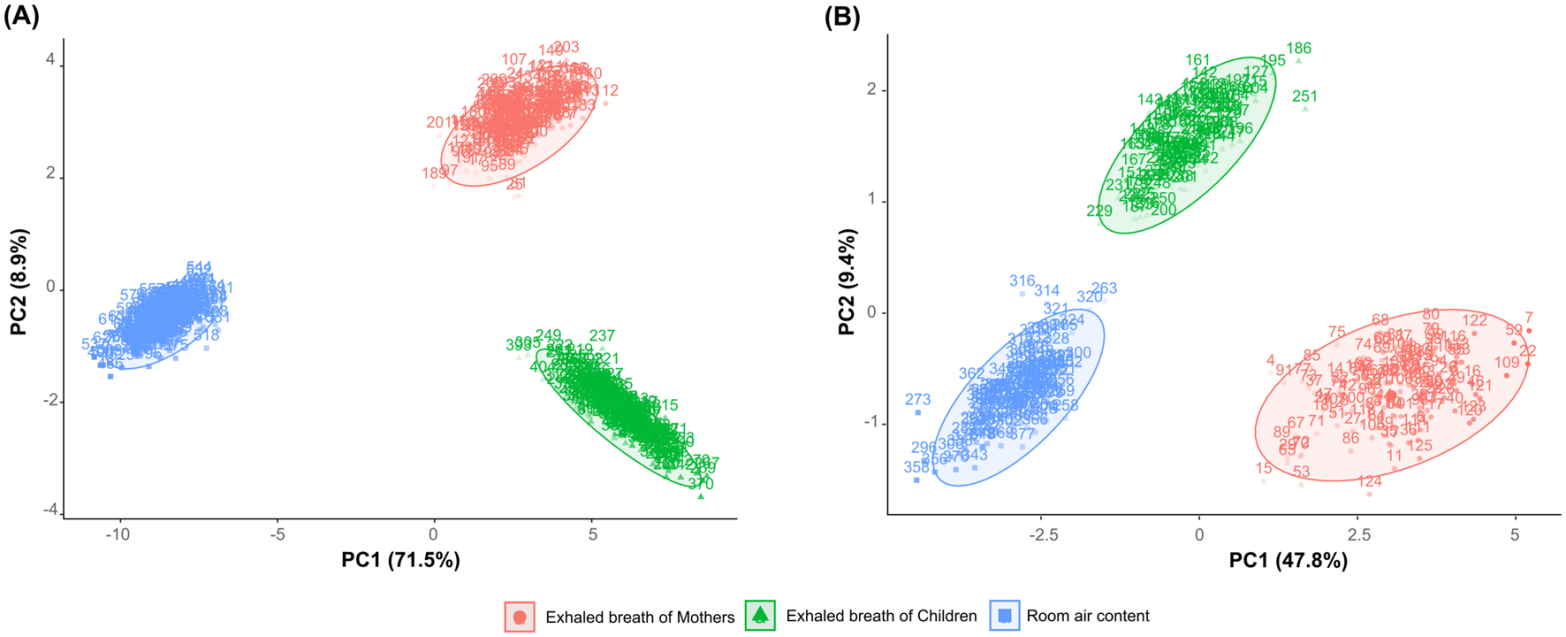
Explorative analysis of filtered features by principal component analysis (PCA). (**A**) PCA score plot (PC-1 vs. PC-2) of breath samples from Group 1. (**B**) PCA score plot (PC-1 vs. PC-2) of breath samples from Group 2.

An exploratory analysis of the filtered features was conducted by principal component analysis (PCA) on both groups of samples. PCA score plots (PC-1 vs. PC-2) are depicted in Fig. 6. The component that represents the maximum percentage of the total variance (PC1) mainly discriminates between the human breath samples and ambient air content samples in both sample groups (Group 1 and Group 2). Moreover, PCA was also performed on features detected by *xcms* (Supplementary Fig. S6).

## Discussion

The preprocessing of data obtained by mass spectrometry has become a bottleneck of exhaled volatilome analysis that is necessary to overcome in the biomarker discovery phase. Indeed, the standardization of digital transformation of data obtained after breath analysis is highly important and essential to move the field forward^12,14,26,39^. Therefore, this paper presents, for the first time, a workflow for preprocessing the data obtained from the analysis of exhaled breath samples of humans using TD-GC/q-MS that integrates the current two main approaches. Although both approaches aim to achieve the same goal, that is, to identify the compounds in the samples, each approach uses a different procedure and time sequence. While features are first determined and then grouped into compounds in the first approach, compounds are first searched for and then the ion profile of the compounds is determined in the second approach.

In addition, most previous studies used proprietary software where the code is not available and the customer has very few configuration options. However, a comprehensive description of the workflow is necessary to achieve a reproducible procedure^40,41^. Accordingly, another advantage of the workflow described herein is the fact that searching for the compounds from breath samples is conducted using open sources. A couple of packages based on the R programming language, *xcms*^23^ and *cliqueMS*^28^, were used in the first approach. In contrast, another R package, *eRah*^27^, was used for the second approach. *CliqueMS* is a newly developed package that was specially designed for data obtained through LC/MS (liquid chromatography–mass spectrometry)^28^. Therefore, this paper demonstrates for the first time that *cliqueMS* can also be successfully used on data obtained by GC/q-MS.

Discrimination between true compounds and duplicate compounds is a great challenge. A high percentage of possible duplicate compounds was observed in both approaches (Fig. 5). Duplication was especially high in the first approach, since *cliqueMS* groups features sample by sample. Thus, the two approaches were connected with double validation using a dot product computation. Only compounds detected by both approaches (dot product greater than 0.7) were selected. In this way, duplicate compounds and identification errors were avoided. The detection of a compound by both strategies is clear evidence of its presence in a breath sample. In fact, the percentage of possible duplicate compounds decreased significantly when the two approaches were connected (Fig. 5). Therefore, the detection of compounds also by *cliqueMS* was useful to achieve a filtered list of compounds obtained by *eRah*. Integration of the two data preprocessing approaches strengthens the validity of the data; in essence it, cross-checks the raw data and reduces errors by eliminating less trustworthy data. Moreover, this connection between methods allowed high accuracy of intensity determination by *xcms* or matching of detected compounds with a spectral library using *eRah*. As a result of this workflow, a matrix was obtained with normalized intensities for the filtered features. This matrix can be used as input data for data analysis. As can be observed, PCA performed with the features obtained by *xcms* (Supplementary Fig. S6) and PCA with the filtered features (Fig. 6) were very similar. Thus, features filtration did not lead to the loss of valuable data. In addition, due to the connection of both strategies, it was possible to determine the features that belong to each compound.

Furthermore, a strategy for filtered compound identification was proposed. These compounds were matched with the NIST spectral library, and two values were calculated: the match factor (provides information about the similarity between spectra) and RI error (provides information about the similarity between retention times). The retention times of compounds from VOCs and linear alkane commercial standards were used as reference compounds to compute the RI error value. In addition, it was possible to study the evolution of the retention times throughout breath sampling by the retention time determination of ubiquitous compounds such as linear alkanes. In this way, ubiquitous compounds of breath samples can be used as internal standards for RI computation.

On the other hand, to our knowledge, it is the first time that exhaled breath from children less than 2 years old has been sampled and analyzed by GC/MS. Simplicity is essential in passive patients such as the pediatric population. For this reason, the strategy was mixed expiratory breath sampling along with collection of ambient air samples. In mixed expiratory breath sampling, the entire air exhaled during breathing (dead space air and alveolar air) is collected without excluding any breath phase. For this reason, mixed expiratory breath sampling is considered to be questionable owing to high contamination from exogenous sources^18^. However, room air content sampling allowed the identification of the background VOCs and minimization of the environmental interference. In addition, it was possible to compare human breath samples and room air samples. The PCA results show that there were differences between the three subsets of breath samples (mothers' exhaled breath, children's exhaled breath, and ambient air). In addition, the difference was greater between the exhaled human breath samples and ambient air than between the exhaled human breath samples (Fig. 6). Therefore, the specimens exhaled by humans included VOCs that emanate from alveolar breath and not only VOCs from the room air.

### Current limitations

Unfortunately, there are very few NIST library entries with RI available. Consequently, the RI error value is not calculated for many compounds. Furthermore, several filtered compounds show close match factor values for a large number of compounds from the NIST library. Therefore, there are over 450 entries in the NIST library with a match factor value between the maximum match factor value found for that compound and the maximum match factor value found for that compound minus 5%. On the other hand, a putative annotation of the compounds rather than a true identification has been performed on most of the filtered compounds^41^. Accordingly, after selecting compounds of interest in statistical analysis, it is highly advisable to use a reference standard for these compounds to confirm their identities.

## Conclusion

A large volume of data is obtained in exhaled volatilome analysis studies aimed at searching for biomarkers. The raw data must be correctly preprocessed to carry out data analysis. Despite the importance of data preprocessing, proprietary software is still used in most studies, which sometimes have little value for certain applications. In this paper, a workflow for the preprocessing of raw data obtained by TD-GC/q-MS is shown for the first time. Herein, free computing tools such as three open language R packages are implemented and cross-checked: *xcms*, *cliqueMS* and *eRah*. This workflow easily/favorably connects two approaches (sample feature detection and sample compound detection) to obtain a matrix useful for data analysis with normalized intensities of features from breath samples. In this sense, integration of both approach allows to reduce duplication of compounds and to obtain a unique list of filtered features and filtered compounds in a short time (2 min per sample). Furthermore, it is clearly shown that the identification of which features belong to each compound and the matching of these compounds with a spectral library are possible as a consequence of the integration of the two approaches. In the future, this process may be simplified in the future with the advancement of free computer-friendly tools and with the increase in available RI values. Overall, we certainly believe that the workflow herein represents a very helpful tool for volatilome analysis implementation as a noninvasive tool for biomarker searching. This will be essential for the development of diagnostic and monitoring strategies for diseases such as asthma, allergies, cancer, etc. Finally, a reproducible protocol for breath sampling from infants is shown in this paper.

## Methods

### Study population

This research is embedded in the NELA (nutrition in early life and asthma) birth cohort study, a population-based birth cohort set up in 2015 in Spain, which recruited mother–child pairs in the 20th week of pregnancy, included in the birth cohort database (2015–03-03—0000–00-00 code). The study was approved by Clinical Research Ethics Committee (CEIC) of the University Hospital Virgen de la Arrixaca of Murcia (Spain). All methods were carried out in accordance with relevant guidelines and regulations. Informed consent was obtained from all subjects aged 18 years and older, and from a parent and/or legal guardian for minors. The inclusion criteria were usual residence in health area I and certain districts of areas VI and VII of the Region of Murcia (Spain); caucasian origin; age between 18–40 years; singleton pregnancy; unassisted conception; and a normal ultrasound scan at 20 weeks of gestation (no major malformations). The NELA study monitors the recruited subjects in 6 phases: 1) at 20 weeks of pregnancy, 2) at childbirth, 3) at 3 months of age, 4) at 18 months, 5) at 4 years and 6) at 7 years. Exhaled breath samples were collected from 3-month-old children and their mothers during the third phase visit. The subjects were distributed into two groups: Group 1 (211 mother–child pairs) and Group 2 (126 mother–child pairs). Breath samples were collected from Group 1 from May 2017 to February 2018, and breath samples were collected from Group 2 from March 2018 to October 2018.

### Breath sampling

Exhaled breath samples were collected from 3-month-old children and mothers of the NELA study using a noninvasive protocol inspired by the approach used by Van der Kant et al*.*^42^. Specifically, mixed expiratory breath sampling^18^ was performed on both mothers and children. Babies, being passive subjects in the sample collection, cannot perform a forced expiration to collect only alveolar air. Therefore, air sampling from the mothers was also without forced expiration. In both cases, exhaled samples are a gas mixture (dead space air and alveolar air). For the mothers, the exhaled air samples were collected in 1 L Tedlar gas sampling bags. For the children, however, the process was performed in two steps: two 400 mL Quintron gas sampling bags, which show lower filling resistence, were filled through a mask, and then the samples were transferred to a Tedlar bag. The air contained in the sample bags was transferred to Tenax tubes to preconcentrate the VOCs (Tenax TA, Markes International). In addition, a room air content sample was taken to assess the possible environmental contaminants using an Easy-VOC syringe (Markes International) that allowed sampling air directly into a third thermal desorption tube. All Tenax tubes were immediately sealed with brass end caps fitted with PTFE ferrules and stored until analysis. If storage period was less than 24 h, the Tenax tubes were stored at room temperature (25 °C). A recent research recommends that exhaled breath samples were kept at cold temperatures (4 °C) for maximum storage stability^43^. Therefore, if storage period was longer than 24 h, tubes were stored at 4 °C. The time taken from sampling to subsequent thermal desorption was always less than a week. The sum of relative intensities of filtered features of 10 samples stored less than 24 h and of 10 samples stored between 5–7 days were compared in order to check the effect of the storage period on the samples (Supplementary Fig. S7). Results of the storage study showed that there were not significant differences due to storage period (*p* value = 0.693). The Tedlar bags were reused 10 times. They were cleaned after each use by washing 5 times with nitrogen gas (99.9% purity). However, the QuinTron bags were not reused. Any material that came into contact with the mother's or child's exhaled air, such as plastic tubes or the mask, was cleaned by spraying with a 70% ethanol solution.

### Analysis of samples by TD-GC/q-MS

Analysis of the exhaled air samples was carried out by a thermal desorption system coupled with gas chromatography-mass spectrometry. Helium was used as the carrier gas. The tube containing the sample was subjected to a two-step thermal desorption (TD) process (UltraTD Multi-Tube Autosampler and Unity Thermal Desorber with Cold Trap, Markes Int. Ltd). The UltraTD was warmed in a Tenax TA tube to 300 °C for 10 min to desorb all its contents. The VOCs from the samples were trapped by carrier gas flowing at 50 mL/min and were loaded onto a Unity cold sorption trap (-10 °C). Then, the cold sorption trap was warmed from -10 °C to 300 °C at 100 °C/s, and the final temperature was held for 10 min. Later, the desorbed VOCs were trapped by helium gas and injected directly into the GC–MS. The system used for sample analysis was a 5977B single quadrupole MS (q-MS) coupled to a 7890B gas chromatograph (GC) from Agilent Technologies with an HP-5 ms Ultra Inert Capillary Column (30 m, 0.25 mm inner diameter, 0.25 µm film thickness). The system used an electron ionization system as the ion source (70 eV). MS data acquisition was performed using full scan mode (scan range of 35–350 AMU). The temperature of the GC column oven was programmed in three temperature ramps: 1) 35 °C for 10 min, 2) increase by 3 °C/min to 121 °C, 3) increase by 20 °C/min to 270 °C, and finally 270 °C for 5 min and increase by 30 °C/min to 300 °C. Once the analysis of the Tenax TA tube contents was completed, the tubes were subjected to a cleaning process. Helium was passed at a flow rate of 20 mL/min for 1 min, and then the tubes were heated to 335 °C in UltraTD for 25 min. This process was repeated twice. Although the breath samples from Groups 1 and 2 were measured with the same methodology, different columns of the same model were used to analyze each group. The n-alkane and VOC calibration standards (Sigma-Aldrich) were loaded onto blank tubes using the same method.

### Statistical analysis

Both the data preprocessing and statistical analysis were conducted using R version 3.5.2. on a 3.6 GHz Intel Core i9 computer. Shapiro–Wilk and Lilliefors test (*nortest* package) were used to test normal distribution of the data. Depending on data distribution, parametric tests (two tailed student's t-test or two tailed ANOVA test followed by Bonferroni post-hoc tests) or non-parametric tests (two tailed Mann–Whitney U test or two tailed Kruskal Wallis test) were performed in order to check if there were significant differences (*p* value < 0.05) in continuous variables. Furthermore, explorative analysis by principal component analysis (PCA) using the package *FactoMineR*^44^ was conducted to observe the differences between human breath samples and room air content samples in both groups of samples (Group 1 and Group 2). The relative intensity values of the filtered features of features obtained by *xcms* were used as input data in the PCA. To avoid introducing systematic biases due to breath sampling, features from pollutants of gas sampling bags (features of N,N-dimethylacetamide and phenol) were not considered in PCA.

## Supplementary Information

Supplementary Information 1.

Supplementary Information 2.
