# Supplementary material for: Data preprocessing workflow for exhaled breath analysis by GC/MS using open sources
Source: Sci Rep. 2020 Dec 15;10:22008. doi: 10.1038/s41598-020-79014-6 (PMC7738550; doi:10.1038/s41598-020-79014-6)
Supplement: Supplementary file 1 — Supplementary Information 1. [file 41598_2020_79014_MOESM1_ESM.pdf]

Data preprocessing workflow for exhaled breath analysis by GC/MS using open sources

Rosa Alba Sola Martínez[1], José María Pastor Hernández[1], Gema Lozano Terol[1], Julia Gallego-Jara[1], Luis García-Marcos[2], Manuel Cánovas Díaz[1] and Teresa de Diego Puente[1]*.

[1] Department of Biochemistry and Molecular Biology and Immunology (B), Faculty of Chemistry, University of Murcia, Campus of Espinardo, Regional Campus of International Excellence ‘‘Campus Mare Nostrum’’, P.O. Box 4021, Murcia E-30100, Spain.

[2] Respiratory and Allergy Units, Arrixaca Children's University Hospital, University of Murcia; and IMIB Biohealth Research Institute; and ARADyAL network, Spain

***Correspondence author:**

Teresa de Diego Puente
Associate Professor.
Biotechnology Group
Dept. of Biochemistry and Molecular Biology B and Immunology
Faculty of Chemistry. University of Murcia.
ResearcherID: N-9215-2013
Orcid ID: 0000-0003-3501-5483
https://scholar.google.com/citations?user=FrGSyt4AAAAJ&hl=en
Phone. +34 868 88 73 95
tdp@um.es

Supplementary Table S1. Time-consuming for data preprocessing and compounds identification of exhaled breath samples from both Group 1 and Group 2.

Samples	Workflow phases	Time-consuming for each phase	Total Time-consuming
**Exhaled breath samples of Mothers from Group 1**	DATA PREPROCESSING	**6 h 42 min**	**7 h 37 min**<br><br>**(2 min 10 s / sample)**
	APPROACH 1	55 min<br>(51 min *xcms* & 4 min *cliqueMS*)	
	APPROACH 2	5 h 54 min	
	DOUBLE VALIDATION	2 min	
	COMPOUNDS IDENTIFICATION	**55 min**	
**Exhaled breath samples of Children from Group 1**	DATA PREPROCESSING	**6 h 17 min**	**7 h 09 min**<br><br>**(2 min 01 s / sample)**
	APPROACH 1	1 h<br>(55 min *xcms* & 5 min *cliqueMS*)	
	APPROACH 2	5 h 16 min	
	DOUBLE VALIDATION	1 min	
	COMPOUNDS IDENTIFICATION	**52 min**	
**Room air content samples from Group 1**	DATA PREPROCESSING	**7 h 13 min**	**8 h 10 min**<br><br>**(2 min 20 s / sample)**
	APPROACH 1	1 h<br>(54 min *xcms* & 6 min *cliqueMS*)	
	APPROACH 2	6 h 9 min	
	DOUBLE VALIDATION	4 min	
	COMPOUNDS IDENTIFICATION	**57 min**	
**Exhaled breath samples of Mothers from Group 2**	DATA PREPROCESSING	**3 h 7 min**	**3 h 58 min**<br><br>**(1 min 53 s / sample)**
	APPROACH 1	38 min<br>(33 min *xcms* & 5 min *cliqueMS*)	
	APPROACH 2	2 h 28 min	
	DOUBLE VALIDATION	1 min	
	COMPOUNDS IDENTIFICATION	**51 min**	
**Exhaled breath samples of Children from Group 2**	DATA PREPROCESSING	**2 h 57 min**	**3 h 47 min**<br><br>**(1 min 49 s / sample)**
	APPROACH 1	38 min<br>(34 min *xcms* & 4 min *cliqueMS*)	
	APPROACH 2	2 h 18 min	
	DOUBLE VALIDATION	1 min	
	COMPOUNDS IDENTIFICATION	**50 min**	
**Room air content samples from Group 2**	DATA PREPROCESSING	**3 h 13 min**	**4 h 5 min**<br><br>**(1 min 56 s / sample)**
	APPROACH 1	38 min<br>(33 min *xcms* & 5 min *cliqueMS*)	
	APPROACH 2	2 h 32 min	
	DOUBLE VALIDATION	2 min	
	COMPOUNDS IDENTIFICATION	**52 min**	

Supplementary Table S2. Parameters of packages for data preprocessing.

	Parameters in xcms	Parameters in cliqueMS	Parameters in *eRah*
**Exhaled breath of Mothers**	**xcmsSet:** method=”matchedFilter”, fwhm=3, max=1000, snthresh=5, step=0.5, steps=1, mzdiff= -0.15<br>**retcor:** method=”obiwarp”, profStep=1<br>**group:** mzwid=0.25, minsamp=10; bw=12 (first part) / 5 (second part)<br>**fillPeaks**	**getCliques:** filter=TRUE, mzerror=0.1, rtdiff=0.1	**Temperature Ramp 1**<br>**deconvolveComp:** min.peak.width=0.653, avoid.processing.mz=c(0), analysis.time=c(1,10), noise.threshold=500<br>**alignComp:** min.spectra.cor=0.85, max.time.dist=29.9, mz.range=c(38:400)<br>**recMissComp:** min.samples=10<br>**Temperature Ramp 2**<br>**deconvolveComp:** min.peak.width=1.1, avoid.processing.mz=c(0), analysis.time=c(10,38.67) , noise.threshold=500<br>**alignComp:** min.spectra.cor=0.85, max.time.dist=7, mz.range=c(38:400)<br>**recMissComp:** min.samples=10<br>**Temperature Ramp 3**<br>**deconvolveComp:** min.peak.width=1, avoid.processing.mz=c(0), analysis.time=c(38.67,46.12), noise.threshold=500<br>**alignComp:** min.spectra.cor=0.85, max.time.dist=7, mz.range=c(38:400)<br>**recMissComp:** min.samples=10
**Exhaled breath of Children**	**xcmsSet:** method=”matchedFilter”, fwhm=3, max=1000, snthresh=5, step=0.5, steps=1, mzdiff= -0.15<br>**retcor:** method=”obiwarp”; profStep=1<br>**group:** mzwid=0.25, minsamp=10, bw=10 (first part) / 4 (second part)<br>**fillPeaks**	**getCliques:** filter=TRUE, mzerror=0.1, rtdiff=0.1	**Temperature Ramp 1**<br>**deconvolveComp:** min.peak.width=0.653, avoid.processing.mz=c(0), analysis.time=c(1,10), noise.threshold=500<br>**alignComp:** min.spectra.cor=0.85, max.time.dist=29.9, mz.range=c(38:400)<br>**recMissComp:** min.samples=10<br>**Temperature Ramp 2**<br>**deconvolveComp:** min.peak.width=1.1, avoid.processing.mz=c(0), analysis.time=c(10,38.67), noise.threshold=500<br>**alignComp:** min.spectra.cor=0.85, max.time.dist=7, mz.range=c(38:400)<br>**recMissComp:** min.samples=10<br>**Temperature Ramp 3**<br>**deconvolveComp:** min.peak.width=1, avoid.processing.mz=c(0), analysis.time=c(38.67,46.12), noise.threshold=500<br>**alignComp:** min.spectra.cor=0.85, max.time.dist=7, mz.range=c(38:400)<br>**recMissComp:** min.samples=10
**Room air content**	**xcmsSet:** method=”matchedFilter”, fwhm=3, max=1000; snthresh=5, step=0.5, steps=1, mzdiff= -0.15<br>**retcor:** method=”obiwarp”, profStep=1<br>**group:** mzwid=0.25, minsamp=10; bw=10 (first part) / 4 (second part)<br>**fillPeaks**	**getCliques:** filter=TRUE, mzerror=0.1, rtdiff=0.1	**Temperature Ramp 1**<br>**deconvolveComp:** min.peak.width=0.653, avoid.processing.mz=c(0), analysis.time=c(1,10), noise.threshold=500<br>**alignComp:** min.spectra.cor=0.85, max.time.dist=29.9, mz.range=c(38:400)<br>**recMissComp:** min.samples=10<br>**Temperature Ramp 2**<br>**deconvolveComp:** min.peak.width=1.1, avoid.processing.mz=c(0), analysis.time=c(10,38.67), noise.threshold=500<br>**alignComp:** min.spectra.cor=0.85, max.time.dist=7, mz.range=c(38:400)<br>**recMissComp:** min.samples=10<br>**Temperature Ramp 3**<br>**deconvolveComp:** min.peak.width=1, avoid.processing.mz=c(0), analysis.time=c(38.67,46.12), noise.threshold=500<br>**alignComp:** min.spectra.cor=0.85, max.time.dist=7, mz.range=c(38:400)<br>**recMissComp:** min.samples=10

Supplementary Table S3. Compounds from the VOC standard with retention index registered in NIST library.

Name of compound	PubChem CID	RI from NIST library	Column 1. RT (min)	Column 2. RT (min)
**Methylene chloride**	6344	391.00	1.84	1.79
**Bromochloromethane**	6333	835.00	3.49	3.41
**Toluene**	1140	764.04	5.13	4.99
**Tetrachloroethene**	31373	808.00	6.88	6.67
**Chlorobenzene**	7964	844.00	9.08	8.80
**Styrene**	7501	892.00	12.66	12.50
**o-Xylene**	7237	891.64	12.80	12.33
**n-Propylbenzene**	7668	952.30	17.38	17.07
**n-Butylbenzene**	7705	1055.98	24.04	23.77
**Naphthalene**	931	1180.70	31.02	30.71

Supplementary Table S4. Linear alkanes retention times observed from each column after n-alkane standard analysis.

Linear alkanes	PubChem CID	RI*	Column 1. RT (min)	Column 2. RT (min)
**Heptane**	8900	700	3.39	3.36
**Octane**	356	800	7.02	6.79
**Nonane**	8141	900	13.44	13.12
**Decane**	15600	1000	20.63	20.36
**Undecane**	14257	1100	26.78	26.55
**Dodecane**	8182	1200	32.23	32.05
**Tridecane**	12388	1300	37.26	37.11
**Tetradecane**	12389	1400	40.58	40.47
**Pentadecane**	12391	1500	42.18	42.09
**Hexadecane**	11006	1600	43.24	43.16
**Heptadecane**	12398	1700	44.08	44.01
**Octadecane**	11635	1800	44.77	44.71
**Nonadecane**	12401	1900	45.40	45.34
**Eicosane**	8222	2000	45.97	45.92

* Kovats retention index

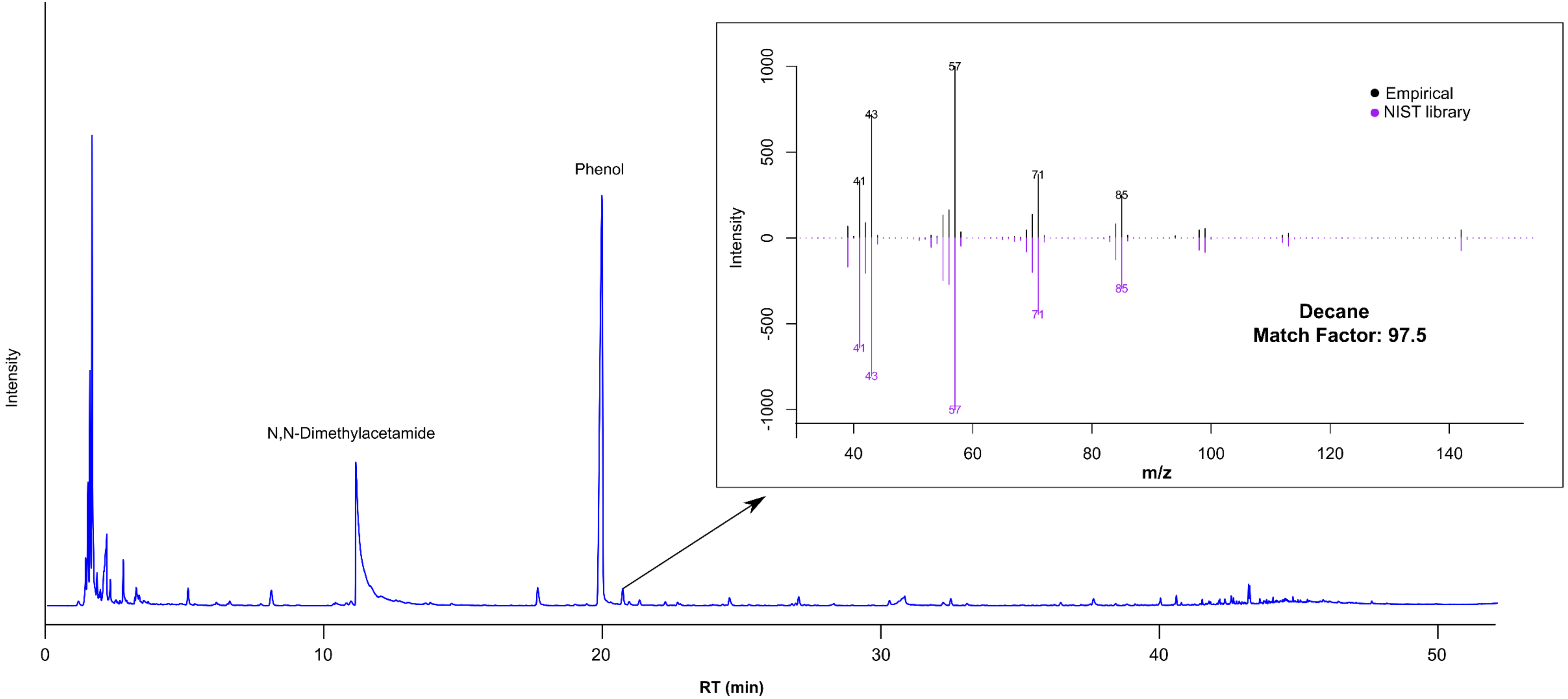

Supplementary Figure S1. Total Ion Chromatogram (TIC) obtained of a breath sample from a mother of Group 1. Contaminants from Tedlar® bags (N,N-Dimethylacetamide and Phenol) can be observed. Spectrum of a compound (Decane) is shown. Extracted spectrum is matched against NIST library. Plots of this figure were generated by *plotChr* and *plotSpectra* functions of package *eRah* in R.

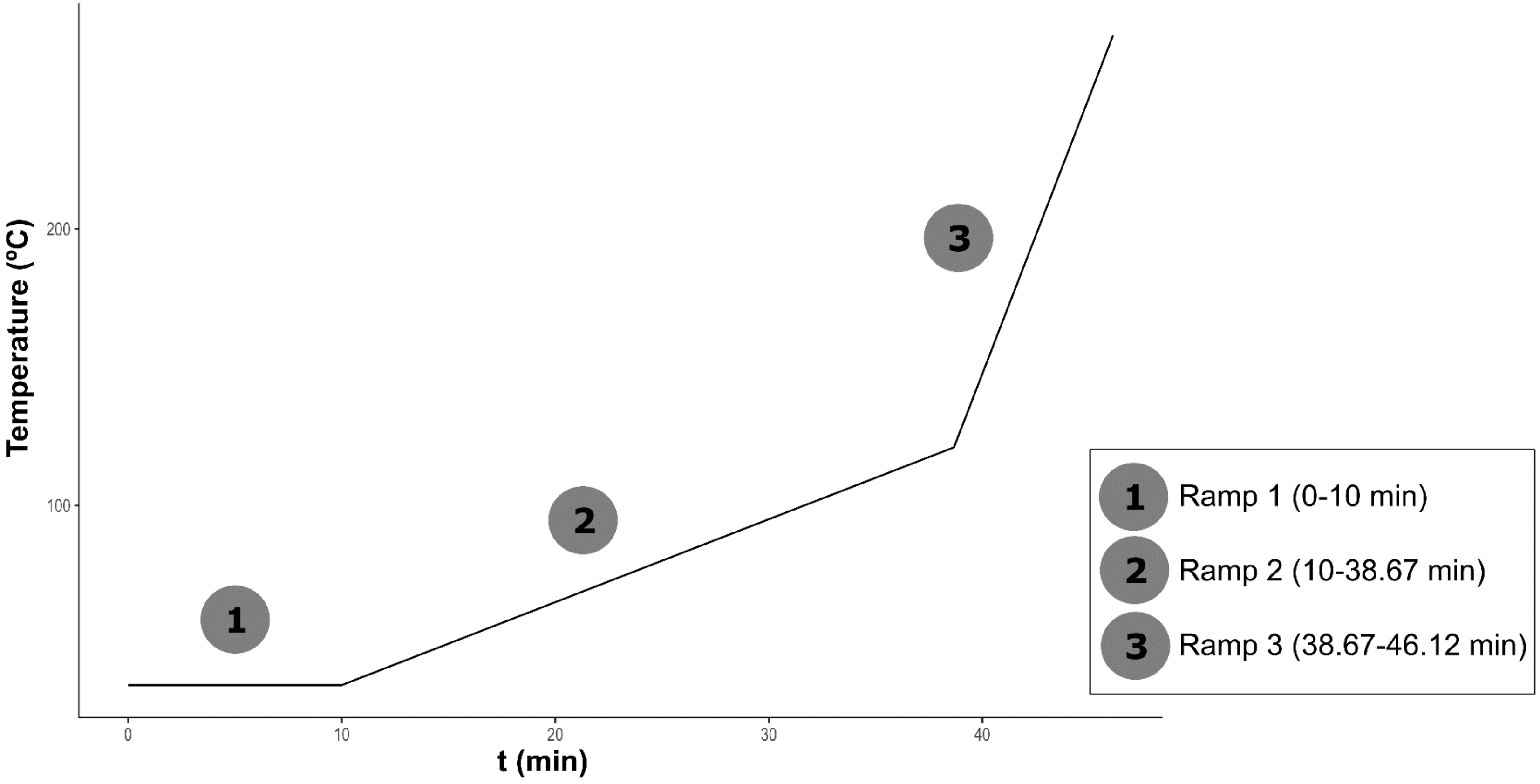

Supplementary Figure S2. Temperature ramps used in exhaled breath analysis by TD-GC/q-MS.

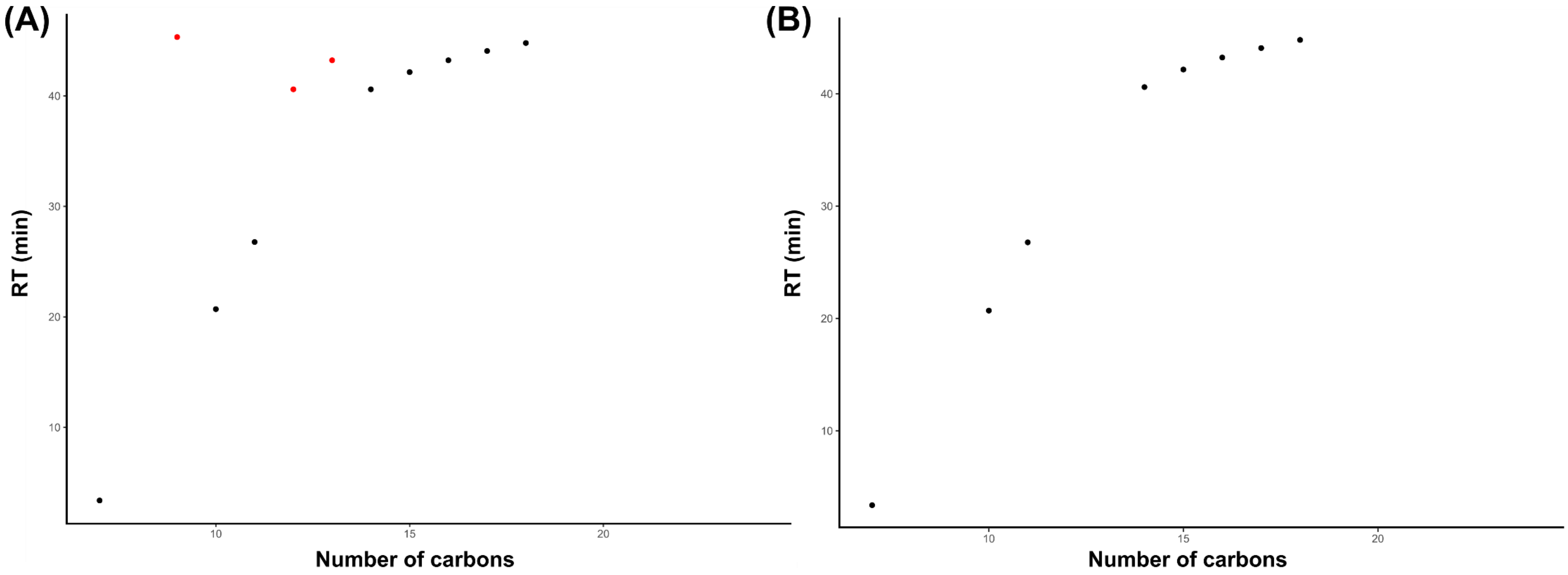

Supplementary Figure S3. Linear alkanes retention times determined in exhaled breath samples. A) Retention times of possible linear alkanes in exhaled breath samples of Group 1. Outliers are highlighted in red. B) Retention times of linear alkanes after removing outliers in exhaled breath of samples of Group 1.

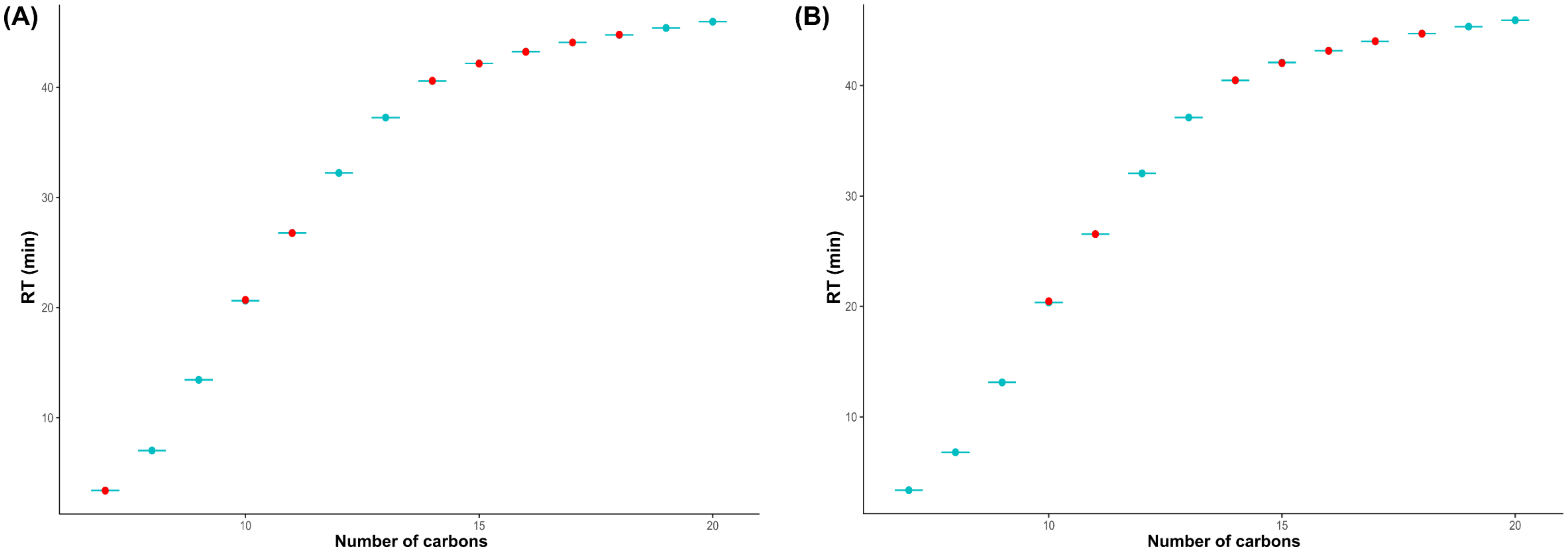

Supplementary Figure S4. Retention times of linear alkanes. A) Linear alkanes retention times determined in exhaled breath samples (red) and linear alkanes retention times observed in commercial standard (blue) in column 1. B) Linear alkanes retention times obtained in exhaled breath samples (red) and linear alkanes retention times observed in commercial standard (blue) in column 2.

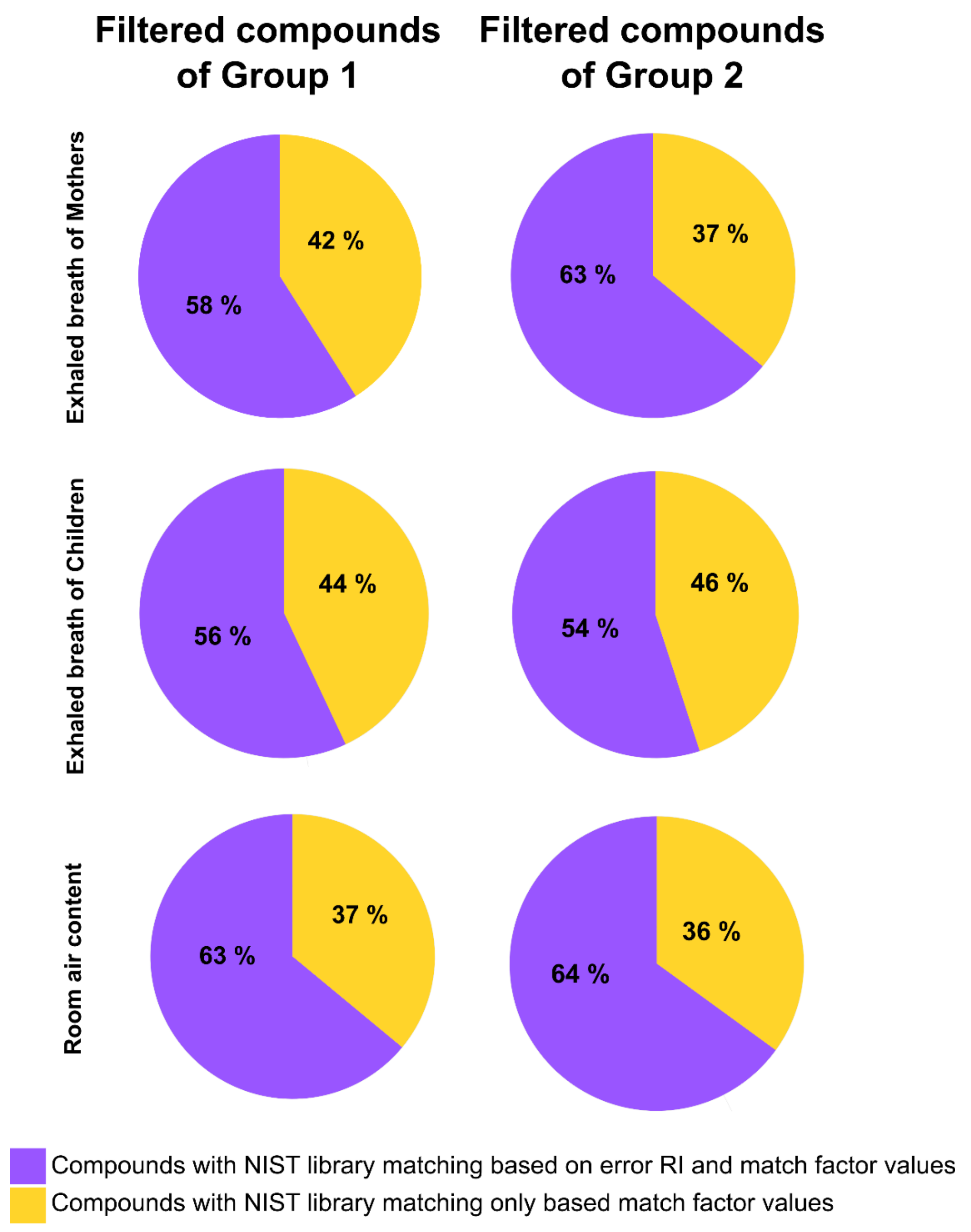

Supplementary Figure S5. Matching between filtered compounds and NIST library.

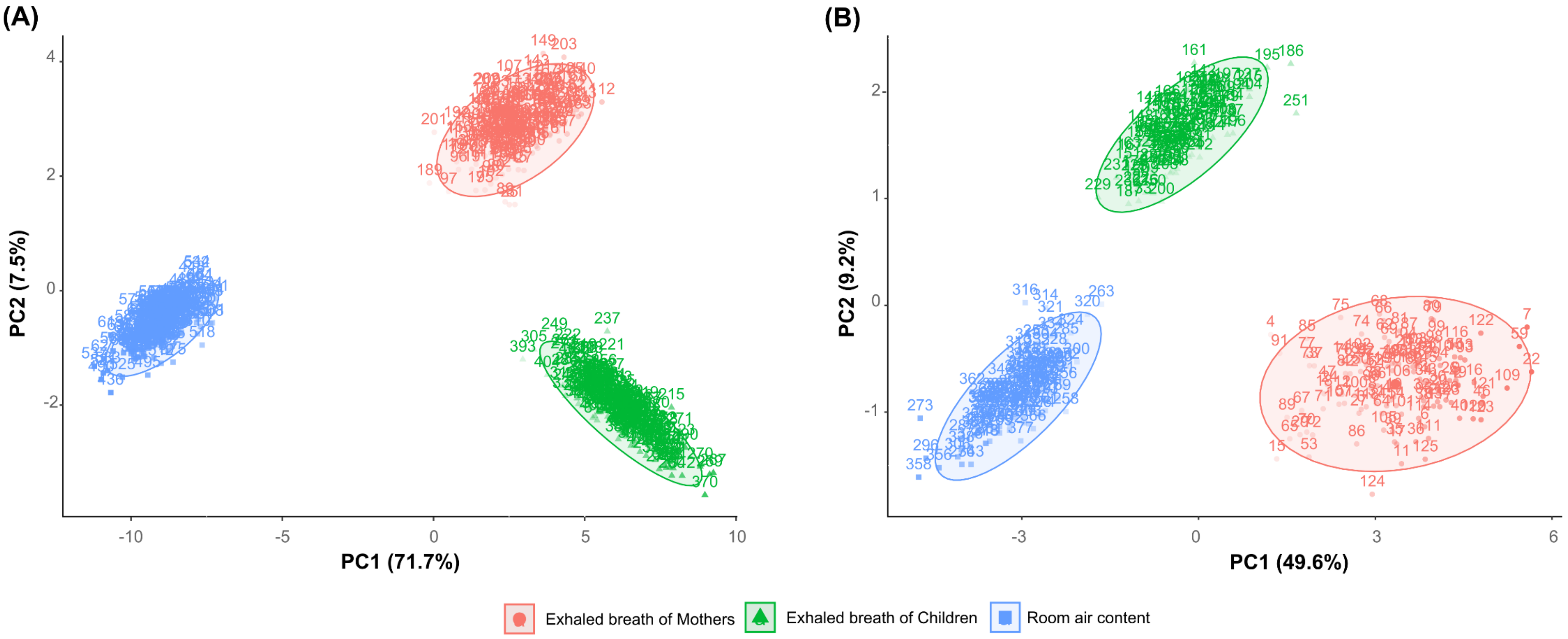

Supplementary Figure S6. Explorative analysis of features obtained by Approach 1 (*xcms* package) by Principal Component Analysis (PCA). A) PCA score plot (PC-1 vs. PC-2) of breath samples from Group 1. B) PCA score plot (PC-1 vs. PC-2) of breath samples from Group 2.

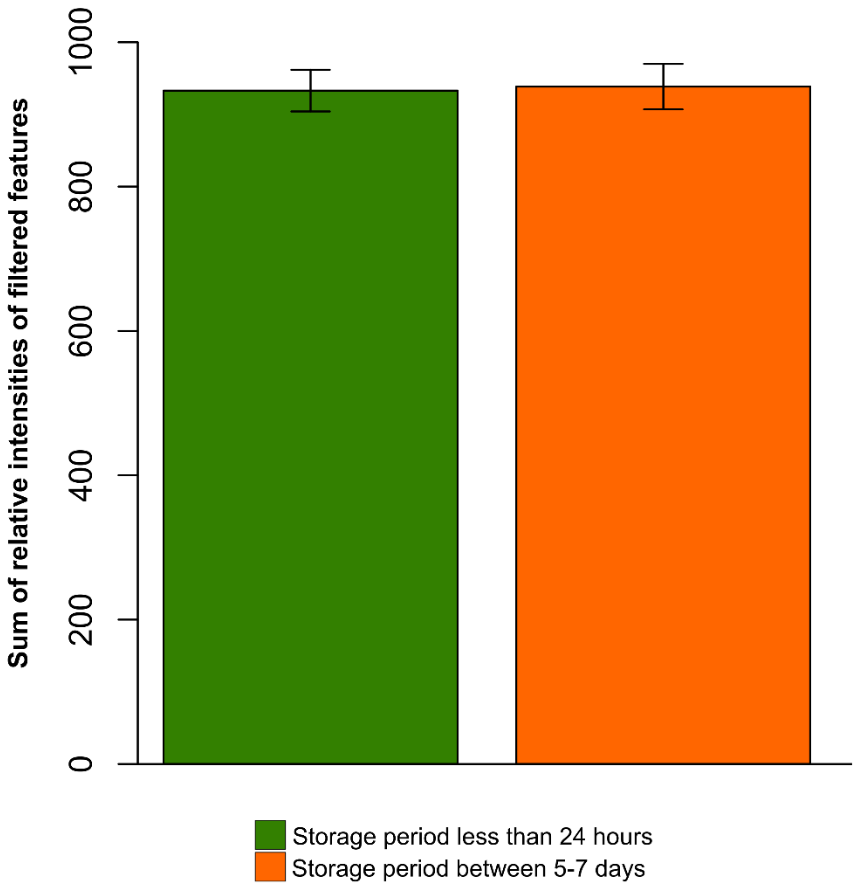

Supplementary Figure S7. Storage study. No significant differences (p-value=0.693) were observed between the sum of relative intensities of filtered features of 10 samples stored less than 24 hours and of 10 samples stored between 5-7 days using two tailed student's test.
