# Supplementary material for: Data preprocessing workflow for exhaled breath analysis by GC/MS using open sources
Source: Sci Rep. 2020 Dec 15;10:22008. doi: 10.1038/s41598-020-79014-6 (PMC7738550; doi:10.1038/s41598-020-79014-6)
Supplement: Supplementary file 2 — Supplementary Information 2. [file 41598_2020_79014_MOESM2_ESM.pdf]

Data preprocessing workflow for exhaled breath analysis by GC/MS using open sources

Rosa Alba Sola Martínez[1], José María Pastor Hernández[1], Gema Lozano Terol[1], Julia Gallego-Jara[1], Luis García-Marcos[2], Manuel Cánovas Díaz[1] and Teresa de Diego Puente[1]*.

[1] Department of Biochemistry and Molecular Biology and Immunology (B), Faculty of Chemistry, University of Murcia, Campus of Espinardo, Regional Campus of International Excellence ‘‘Campus Mare Nostrum’’, P.O. Box 4021, Murcia E-30100, Spain.

[2] Respiratory and Allergy Units, Arrixaca Children's University Hospital, University of Murcia; and IMIB Biohealth Research Institute; and ARADyAL network, Spain

***Correspondence author:**

Teresa de Diego Puente

Associate Professor.

Biotechnology Group

Dept. of Biochemistry and Molecular Biology B and Immunology

Faculty of Chemistry. University of Murcia.

ResearcherID: N-9215-2013

Orcid ID: 0000-0003-3501-5483

https://scholar.google.com/citations?user=FrGSyt4AAAAJ&hl=en

Phone. +34 868 88 73 95

tdp@um.es

Supplementary Tutorial. Data preprocessing workflow for volatilome analysis by GC/q-MS. R-code of the main functions.

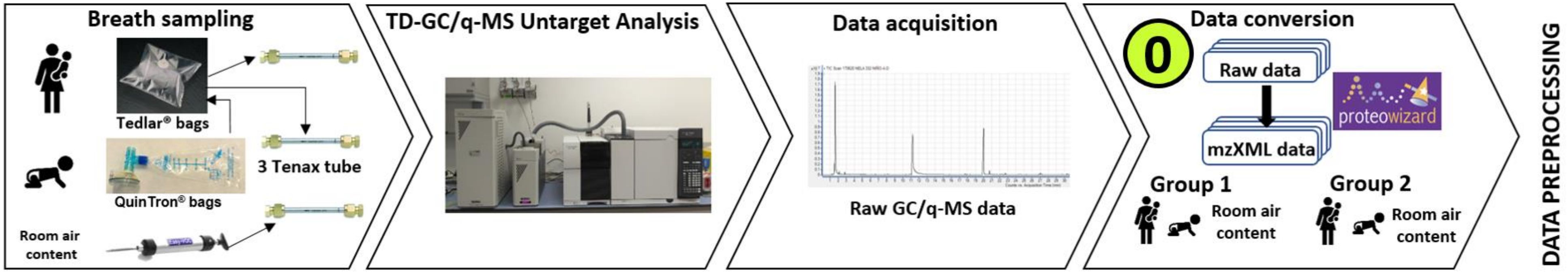

0 Inputdata

Convert MS raw files into open **mzXML** data format

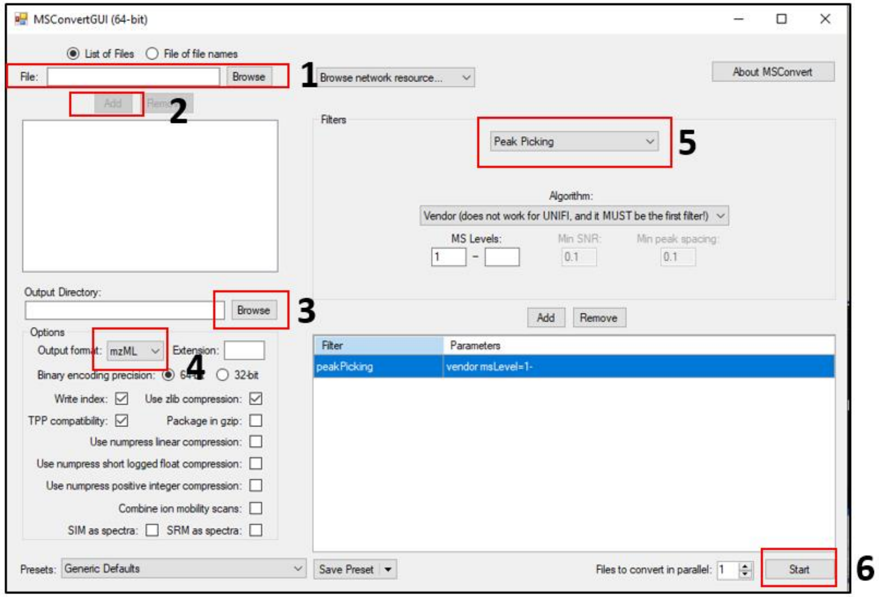

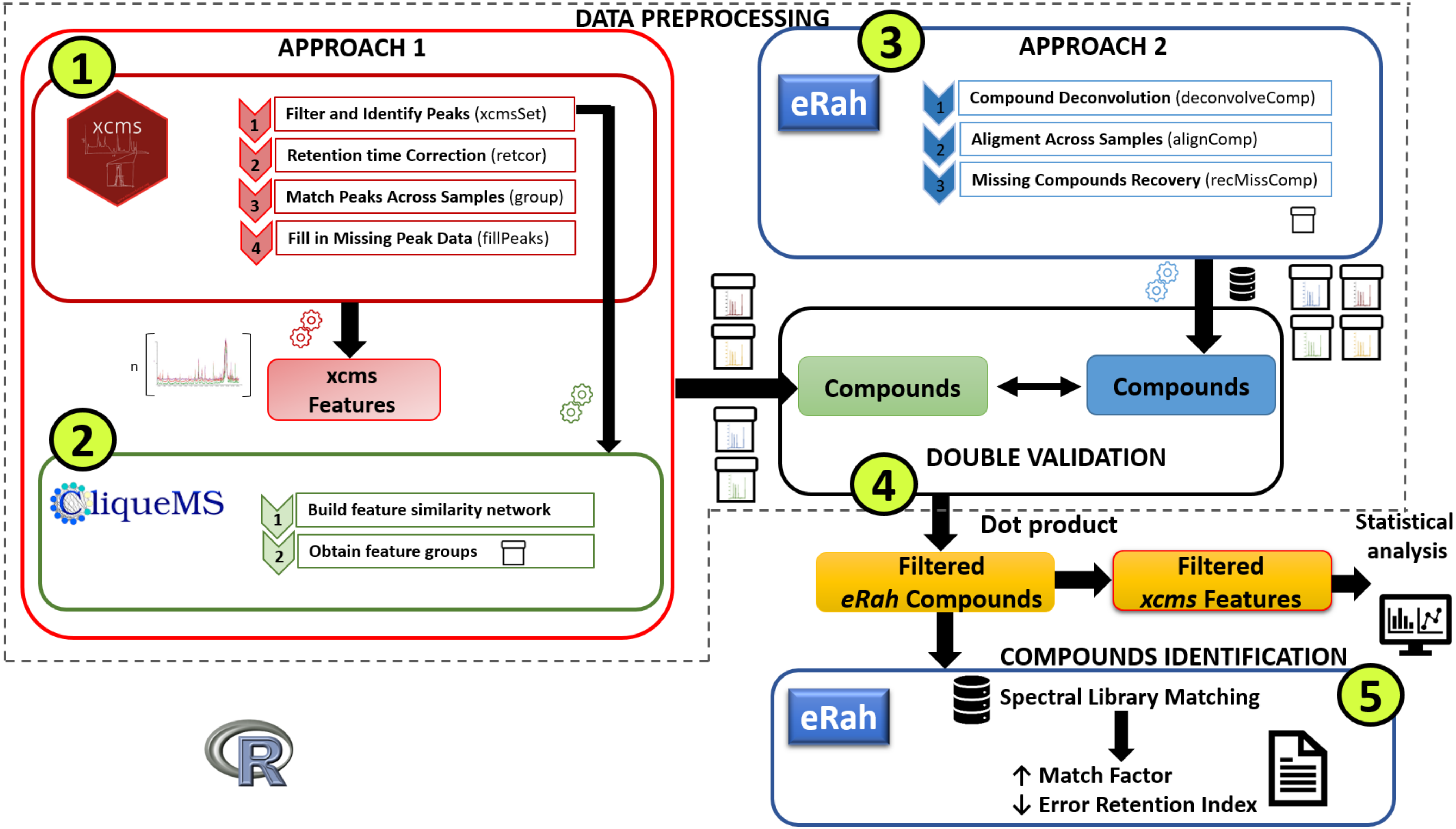
DATA PREPROCESSING
APPROACH 1
1
xcms
1 Filter and Identify Peaks (xcmsSet)
2 Retention time Correction (retcor)
3 Match Peaks Across Samples (group)
4 Fill in Missing Peak Data (fillPeaks)
n
xcms Features
2
CliqueMS
1 Build feature similarity network
2 Obtain feature groups
3
APPROACH 2
eRah
1 Compound Deconvolution (deconvolveComp)
2 Aligment Across Samples (alignComp)
3 Missing Compounds Recovery (recMissComp)
Compounds
Compounds
DOUBLE VALIDATION
4
Dot product
Filtered eRah Compounds
Filtered xcms Features
Statistical analysis
COMPOUNDS IDENTIFICATION
5
eRah
Spectral Library Matching
↑ Match Factor
↓ Error Retention Index

1 APPROACH 1 (*xcms* package)

1 **Filter and Identify Peaks** (xcmsSet)

```
bpparam <- BiocParallel::SnowParam(workers = 5)
xcms.out1 <- xcmsSet(files = inputdata, method = "matchedFilter", fwhm = 3,
             max = 1000, snthresh = 5, step = 0.5, steps = 1, mzdiff=
             -0.15, BPPARAM = bpparam)
```

2 **Retention time Correction** (retcor)

```
xcms.out2 <- retcor(xcms.out1, method="obiwarp",profStep=1)
```

3 **Match Peaks Across Samples** (group)

```
xcms.out3 <- group(xcms.out2,mzwid=0.15,minsamp=10,bw=#)
```

4 **Fill in Missing Peak Data** (fillPeaks)

```
xcms.out4 <- fillPeaks(xcms.out3,BPPARAM = bpparam)
```

Search parameter value in Supplementary Table S2

Smith, C. A., Want, E. J., O'Maille, G., Abagyan, R. & Siuzdak, G. XCMS: Processing Mass Spectrometry Data for Metabolite Profiling Using Nonlinear Peak Alignment, Matching, and Identification. *Anal. Chem.* **78**, 779–787 (2006).

2 APPROACH 1 (*cliqueMS* package)

CliqueMS

1. Build feature similarity network
2. Obtain feature groups

```
cliqueMS.out1 <- getCliques(xcms.out1[,i], filter = TRUE, mzerror=0.1,
                   rtdiff=0.1)

cliqueMS.out2 <- getIsotopes(cliqueMS.out1, ppm = 500)

cliqueMS.out3 <- cliqueMS.out2$peaklist
```

i cliqueMS annotates samples one by one

Senan, O. *et al.* CliqueMS: A computational tool for annotating in-source metabolite ions from LC-MS untargeted metabolomics data based on a coelution similarity network. *Bioinformatics* **35**, 4089–4097 (2019).

3 APPROACH 2 (*eRah* package)

eRah

```
erah.out <- newExp(instrumental = "inst.csv", phenotype = "pheno.csv")
```

1 **Compound Deconvolution** (deconvolveComp)

```
dec.par <- setDecPar(min.peak.width = #, avoid.processing.mz = c(0),
                analysis.time = #)

erah.out <- deconvolveComp(erah.out, dec.par)
```

Search parameter value in Supplementary Table S2

Domingo-Almenara, X. *et al.* eRah: A Computational Tool Integrating Spectral Deconvolution and Alignment with Quantification and Identification of Metabolites in GC/MS-Based Metabolomics. *Anal. Chem.* **88**, 9821–9829 (2016).
Domingo-Almenara, X. *An introduction to erah package*. https://cran.r-project.org/web/packages/erah/vignettes/eRahManual.pdf (2020).

3 APPROACH 2 (*eRah* package)

eRah

Aligment Across Samples (alignComp)

```
al.par <- setAlPar(min.spectra.cor = 0.85, max.time.dist =#,mz.range = c(38:400))

erah.out <- alignComp(erah.out, alParameters = al.par, blocks.size = 55)
```

Missing Compounds Recovery (recMissComp)

```
erah.out <- recMissComp(erah.out, min.samples = 10)
```

Search parameter value in Supplementary Table S2

Domingo-Almenara, X. *et al.* eRah: A Computational Tool Integrating Spectral Deconvolution and Alignment with Quantification and Identification of Metabolites in GC/MS-Based Metabolomics. *Anal. Chem.* **88**, 9821–9829 (2016).
Domingo-Almenara, X. *An introduction to erah package*. https://cran.r-project.org/web/packages/erah/vignettes/eRahManual.pdf (2020).

4 DOUBLE VALIDATION

```
dotproduct <- function(x,y, from=38, to = 450){
  mzrange <- seq(from =from, to = to, by=1)
  x2 <- sapply(mzrange,function(i){
    dif <- abs(round(x[,1])-i)
    res <- 0
    if(any(dif<0.5)){
      res <- x[which(dif==min(dif)),2]
    }
    return(res)
  })
  y2 <- sapply(mzrange,function(i){
    dif <- abs(round(y[,1])-i)
    res <- 0
    if(any(dif<0.5)){
      res <- y[ which(dif==min(dif))  ,2]
    }
    return(res)
  })
  as.vector((x2 %*% y2) / (sqrt(sum(x2^2)) * sqrt(sum(y2^2))))
}
```

x: vector with relative intensities of *xcms-cliqueMS* compounds

y: vector with relative intensites of *eRah* compounds

5 COMPOUNDS IDENTIFICATION (*eRah* package)

eRah

Spectral Library Matching & Match factor computation

```
ident.out <-identifyComp(erah.out, id.database = NIST_library, n.putative = 450)
```

Error Retention Index computation

```
errorRI.out<-computeRIerror(ident.out, id.database = NIST_library, reference.list = referenceCompounds, ri.error.type = "relative",plot.results = F)
```

Linear alkanes and compounds from the VOC standard have been used as reference compounds (Supplementary Table S3 and Supplementary Table S4)

Domingo-Almenara, X. *et al.* eRah: A Computational Tool Integrating Spectral Deconvolution and Alignment with Quantification and Identification of Metabolites in GC/MS-Based Metabolomics. *Anal. Chem.* **88**, 9821–9829 (2016).

Domingo-Almenara, X. *An introduction to erah package*. https://cran.r-project.org/web/packages/erah/vignettes/eRahManual.pdf (2020).
